# Data on Investigating the resilience of female smallholder livestock farmers to agricultural drought in South Africa's Northern Cape Province

**DOI:** 10.1016/j.dib.2023.109780

**Published:** 2023-11-07

**Authors:** Lindie von Maltitz, Yonas T. Bahta

**Affiliations:** Department of Agricultural Economics, Faculty of Agricultural and Natural Sciences, University of the Free State, P.O. Box 339, Bloemfontein 9300, South Africa

**Keywords:** Risk management, Women in agriculture, Gender bias, Empowerment, Climate extremes

## Abstract

The resilience to agricultural drought of female smallholder livestock farmers in the Northern Cape province of South Africa was examined using the Abbreviated Women's Empowerment in Agriculture Index (A-WEAI). The A-WEAI consists of the 5DE index (five domains of empowerment) and the GPI (gender parity index). The total population interviewed was 154 participants (61 women in long-term relationships plus their male partners and 32 women who are single, divorced, or widowed). Researchers and policymakers can use the dataset to identify critical areas that need to be addressed to enhance women's empowerment in agriculture, which will increase their resilience to climate extremes such as agricultural drought.

Specifications Table

**Table:** 

Subject	Agricultural Economics
Specific subject area	Enhancing the resilience of female smallholder livestock farmers to agricultural drought through empowerment
Data format	Raw, Analyzed, Filtered, xls file (dataset with labels)
Type of data	Household survey
Data collection	A multi-stage sampling was used. In the first stage, the Northern Cape Province of South Africa was selected as it was identified as one of the country's main livestock-producing provinces. The impact of drought is also a characteristic that could be researched in this province, as it was declared a drought disaster area in 2018 by the South African Government. In the second sampling stage, the four local district municipalities of the Frances Baard District of the Northern Cape were chosen randomly. These are Phokwane, Sol Plaatje, Magareng and Dikgatlong. The respondents' sample was drawn from female smallholder livestock farmers registered for assistance at the Department of Agriculture, Forestry and Fisheries (DAFF). This list contained a total of 127 female farmers who farmed mainly with livestock. Random sampling was used to select participants for the study from this list. A semi-structured questionnaire was used and administered face-to-face by the principal researchers and trained enumerators. The Abbreviated Women's Empowerment in Agriculture Index (A-WEAI) uses a prescribed questionnaire that is adjusted according to the specific circumstances of the research area. Interviews were conducted with participants. Data was entered and processed in Excel. Once the empowerment status of women was calculated, information collected with a structured questionnaire but not included in the calculation of the index, namely the age of the respondent, marital status of the respondent, years of farming experience of the respondent, and level of education of respondent was correlated to empowerment status using Pearson's chi-coefficient. To place results into the context of the Northern Cape Province of South Africa, reflective dialogue was recorded during the process of data collection.
Data source location	The data is stored at the University of the Free State.
Data accessibility	Repository name: Figshare Data identification number : 10.38140/ufs.24032229 Direct URL to data https://ufs.figshare.com/articles/dataset/Data_on_the_resilience_of_female_smallholder_livestock_farmers_xlsx/24032229
Related research article	Bahta, Y.T., von Maltitz, L. (2021). Empowerment of smallholder female livestock farmers and its potential impacts to their resilience to agricultural drought. AIMS Agriculture and Food 6(2) 603-630. 10.3934/agrfood.2021036

## 1 Value of the Data

• The data presented assists in understanding the unique challenges female smallholder farmers experience.
• By observing agricultural drought resilience through a gender perspective, information is presented for policies and programs aimed at assisting and policy intervention mechanisms.
• Policymakers and government departments may benefit from the presented data to assist them in formulating appropriate solutions and assistance to challenges.
• Researchers can benefit from the data by using it to expand research on gender in agriculture and climate change in general and agricultural drought in particular.
• It provides the potential to duplicate similar studies related to empowerment, which is measured by the Abbreviated Women's Empowerment in Agriculture Index (A-WEAI), five domains of empowerment (5DE) [production, resources, income, leadership, and time] and the gender parity within the household (GPI).

## 2 Data Description

Data was collected and categorized according to the Abbreviated Women's Empowerment in Agriculture Index (A-WEAI) specifications. It comprises two sub-indexes, namely the five domains of empowerment (5DE) [production, resources, income, leadership, and time] and the gender parity within the household (GPI) [1]. Data was captured and processed according to the requirements of the different indices using Microsoft Excel. The prescribed weights of the 5DE and GPI sub-indexes are 90 % and 10 % respectively. The selection of these weights was based on the fact that more emphasis is placed on the 5DE in the index, but gender equality is also recognized [2]. Then, adapted from a strategy used by the Enhancing Opportunities for Women's Enterprises program (EOWE) in Vietnam, namely a Social and Behavior Change Communication (SBCC) strategy to aid in the reporting of restraining gender norms and other empowerment issues, was used to put results into context and applied in this study [3,4]. It involves sharing dialogue and experiences to positively influence change in communities. Lastly, the correlation between empowerment status and other factors (such as age, educational level, marital status, and farming experience) was also calculated using Pearson's chi-square. Statistical significance of the correlation between factors not included in the A-WEAI was calculated and reported on.

Table 1 provides the domains, indicator names, survey questions involved, aggregation method, inadequacy cut-off, and weight of each indicator used.

**Table 1:** Format of data collected.

Dimension	Indicator Name	Survey question examples	Aggregation method	Inadequacy cut-off	Weight
Production	Input in productive decisions	How much input did you have in making decisions about livestock? Do you feel that you can make your own decisions regarding this activity?	Achievement in two	Inadequate if the individual participates BUT does not have at least some input into decisions, does not make the decisions, or feels that she could make the decisions if she wanted to	1/5
Resources	Ownership of assets Access to and decisions on credit	Does anyone in the household have any [ITEM]? Do you own any of them? (Livestock, equipment, etc.) Has anyone in your household taken any loans or borrowed cash from anywhere in the past 12 months? Who decided to borrow? How much input do you have in deciding how to use the funds?	Achievement in any, if not only, small assets (chickens, non-mechanized equipment, and small consumer durables) Achievement in any	Inadequate if the household does not own any asset or if the household owns the type of asset BUT she/he does not own most of it alone. Inadequate if the household has no credit OR used a source, but she/he did not participate in any decisions about it	2/15 1/15
Income	Control over the use of income	How much input did you have in decisions on using income generated in the household? Do you feel that you can make your own decisions regarding income?	Achievement in any, if not only, minor household expenditures	Inadequate if participated in activity BUT has no input or little input in decisions about the income generated	1/5
Leadership	Group membership	Are you a member of any agricultural/religious/business Group?	Achievement in any	Inadequate if not part of at least one group	1/5
Time	Workload	Worked more than 10.5 hours in the previous 24 hours	N/A	Inadequate if works more than 10.5 hours a day	1/5

Source: Author adapted from [3]

**Fig. 1:**
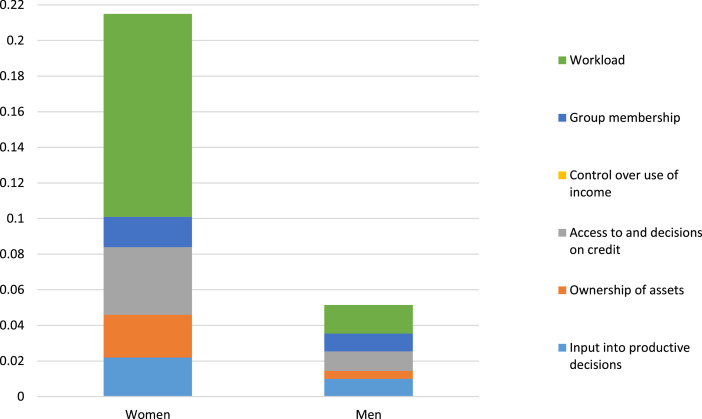
The contribution of each indicator to the disempowerment of women and men.

Source: Authors compilations based on the surveyed data

**Fig. 2:**
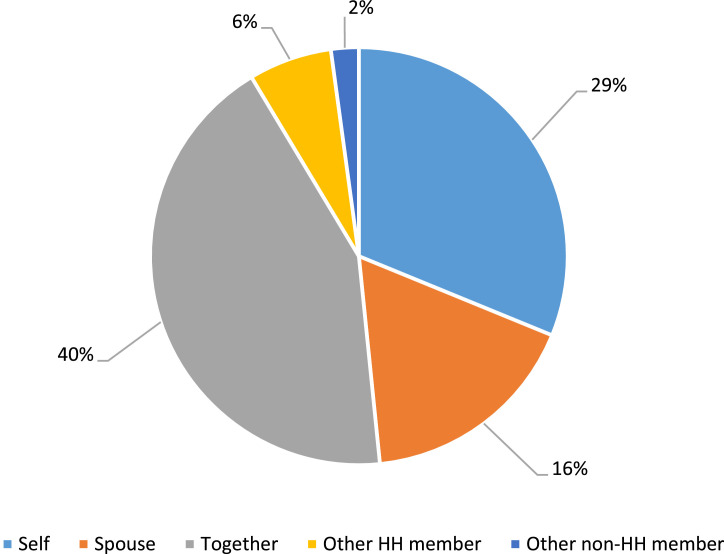
Decision-maker with regards to livestock production in dual-gender households.

Source: Authors compilations based on the surveyed data

**Fig. 3:**
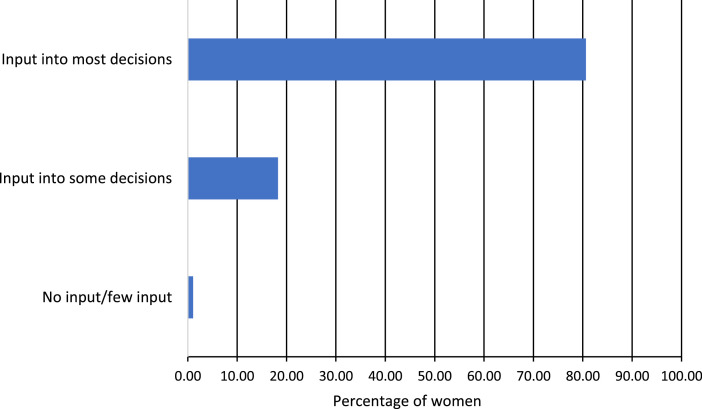
Level of input into the above decision-making.

Source: Authors compilations based on the surveyed data

**Fig. 4:**
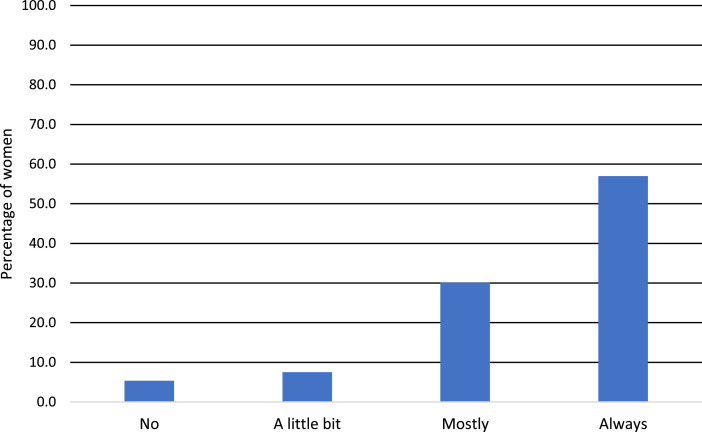
Decisions with regard to livestock production.

Source: Authors compilations based on the surveyed data

**Fig. 5:**
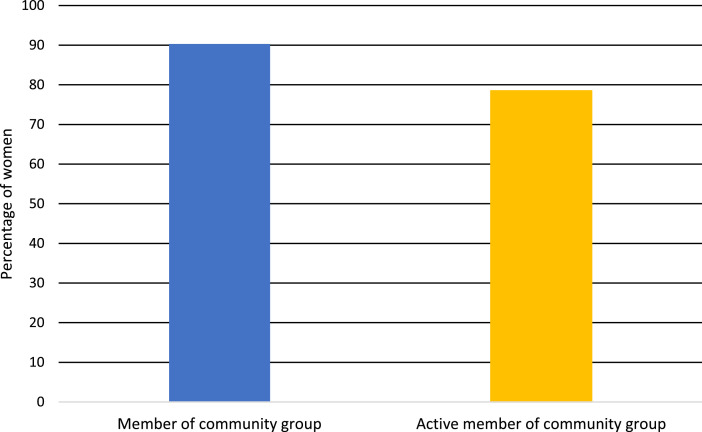
Membership in a community group.

Source: Authors compilations based on the surveyed data

Table 2 and Fig. 1, Fig. 2, Fig. 3, Fig. 4, Fig. 5 present the summary of key indicators. As indicated in Table 2, the five domains of the empowerment index are calculated by considering a prescribed aggregation method that implicates the adequacy of empowerment in a certain domain. Table 2 shows the A-WEAI index result.

**Table 2:** The 5DE decomposed by dimension and indicator for the survey sample.

Statistics	Production	Resources		Income	Leadership	Time
	Input into productive decisions	Ownership of assets	Access to and decisions on credit	Control over the use of income	Group membership	Workload
Indicator weight	0.2	0.13	0.067	0.2	0.2	0.2
Women						
Censored headcount	0.108	0.18	0.570	0	0.086	0.570
% contribution	10 %	11.3 %	17.7 %	0	8 %	52.97 %
Absolute contribution	0.022	0.024	0.038	0	0.017	0.114
Men						
Censored headcount	0.049	0.033	0.164	0	0.049	0.082
% contribution	19.1 %	8.5 %	21.4 %	0	19.13 %	31.89 %
Absolute contribution	0.010	0.004	0.011	0	0.010	0.016
Percentage of women lacking gender parity (HGPI)		Average empowerment gap (*I_GPI_*)		Gender Parity Index (GPI)		
50.82		17.35		0.9118		

Source: Author's calculations based on surveyed data

Fig. 1 and Table 2 illustrate that the dimensions contributing the most to the disempowerment of female smallholder livestock farmers are workload (52.97 %) and access to and decisions on credit (17.7 %). Women have control or at least some form of input into the use of their own/their family's income, but lack of ownership of assets (11.3 %), lack of input into productive decisions (10 %), and lack of group membership (8 %) also contribute to their disempowerment.

The main contributors to the disempowerment of men are also workload (31.89 %) and access to and decisions on credit (21.4 %) whilst lack of group membership (19.13 %) and lack of input into productive decisions (19.1 %) also play a significant role.

The GPI index indicates that 50.82 % of women lack gender parity in their households (HGPI), with an average empowerment gap of 17.35 % (IGPI). The GPI for the research area is 0.9118, indicating that most households enjoy relative gender parity (Table 2). The results contribute significantly to policy formulation that deals with women's empowerment and, more specifically, female smallholder livestock farmers. Fig. 1 depicts the contribution of each indicator to the disempowerment of women and men.

In order to assess the “inputs into productive decisions,” the following questions were asked: “Who normally makes decisions with regard to the specific farming activity? Was it Self, spouse, together, another household member, or another non-household member? How much input did you have in the decisions made? No input, some input, or sufficient input? Do you feel that you can make your own personal decisions with regard to the said activity? No, a little bit, mostly, always? Achievement was allocated when the individual had sufficient achievement in at least two of the questions. The individual was considered disempowered if she participated in the activity but did not have at least some input into the decision-making process. The results are summarized in Fig. 2.

The above results in Fig. 2 indicate that in most of the cases, decision-making occurred as a team, male and female, in the household (40 %). Women had the freedom to make decisions (29 %) concerning the livestock enterprises on their own, and 16 % of the women reported that they had no input and their spouses/partners made the decisions in this regard, 6 % of the respondents reported that other household members, such as a father or a son, made the decisions, and 2 % indicated that decisions are made by a non-household member, such as relatives who own the livestock but live somewhere else.

The level of input that female farmers felt they had in decision-making is illustrated in Fig. 3 below. The majority of the women (80.6 %) indicated sufficient input into productive decisions, while 18.3 % indicated that they had some input into decisions, and only 1.1 % of the women said that they had no input into productive decision-making. When the men were interviewed, 98.4  % indicated they had enough input into decision-making in the household, while the rest (1.6 %) said they did not. In this case, the women's answers mostly agreed with the men's. This implies that encouraging teamwork between household males and females would improve the household's collective resilience to agricultural drought.

Fig. 4 indicates that the majority of the women (57 %) always experience freedom in their households to actively participate in the decision-making processes with regard to livestock production, 30.1 % mostly experience freedom in their households to participate in the decision-making processes, 7.5 % indicated a little bit freedom in their households to participate in the decision-making processes. The minority (5.4 %) said they were manipulated/dominated by their spouse, other household members, or other non-household members. This implies that limitation on decision-making power has a negative impact on resilience against climate extremes such as drought. Decision-making power is not confined to household-level but extended to governmental influence, which can also play a significant role, especially in South Africa, where decisions are often forced onto beneficiaries by different government and municipal departments due to the fact that assets (such as land and water) are in many cases controlled by them.

As shown in Fig. 5, of the 93 women interviewed, 84 (90 %) belonged to some form of a community group, be it church/religion based, AFASA (African Farmers Association of South Africa), a producers group, etc. Out of 93 women interviewed, 78 % were active members in the community groups by attending meetings, serving on committees, etc.

Regarding importance, a lack of group membership contributed 8 % (Table 2) to the disempowerment of the women interviewed. The A-WEAI considers this because it considers group membership a resource in terms of information and support. This implies the significance of the role of group membership in resilience against climate shocks such as drought.

## 3 Experimental Design, Materials and Methods

In this study, multi-stage sampling was used. In the first stage, the Northern Cape Province of South Africa was selected as it was identified as one of the country's main livestock-producing provinces. The impact of drought is also a characteristic that could be researched in this province, as it was declared a drought disaster area by the South African Government [5]. In the second sampling stage, the four local district municipalities of the Frances Baard District of the Northern Cape were chosen randomly. These are Phokwane, Sol Plaatje, Magareng and Dikgatlong. The respondents' sample was drawn from female smallholder livestock farmers registered for assistance from the Department of Agriculture, Forestry and Fisheries (DAFF). This list contained a total of 127 female farmers that farmed mainly with livestock. Random sampling was used to select participants for the study from this list.

Specific requirements for participants of the study were:

­ Respondents had to be female smallholder farmers farming primarily with livestock and willing to participate in the research study.
­ Respondents had to reside in the Frances Baard District Municipality of the Northern Cape as this was the area identified to be important in terms of livestock production.
­ In order to calculate the gender parity index (GPI) of the study area, the focus was on married women or women in a permanent relationship with men who are also actively involved in the farming operation. Data was, however, also collected from single, divorced, and widowed women. Using the simple random selection formula, a total of 93 female smallholder livestock farmers were interviewed, 61 (66 %) of them were either married or in a permanent relationship. Their male partners (61) were also interviewed using the same questionnaire as the one used for the women.
­ The total population interviewed was therefore 154 participants (93 female smallholder livestock farmers (61 women in long-term relationships plus their male partners (61)) plus 32 women who are single, divorced, or widowed).

A questionnaire was structured according to the instructional guide on the Abbreviated Women's Empowerment in Agriculture Index and completed through interviews. After the data was collected, it was captured on Excel and cleaned immediately after capturing. The necessary calculations were done using the allocated weights, and an inadequacy score was calculated for each participant.

The sample size determined based on list of female smallholder livestock farmers provided by the Department of Agriculture in the Northern Cape who seek assistance from the governemt during the agricultural drought. A total of 127 women were listed as primarily livestock farmers. From 127 female smallholder livestock farmers, 86 were chosen using the simple random sampling formula of Cochran [6] and Bartlett et al. [7] . The correct sample size was determined using Cochran's [6] sample size formula Eq. (1):

(1) $$N_{0}=\frac{{\left(t\right)}^{2*}\left(p\right)\left(q\right)}{{\left(d\right)}^{2}}$$

Where, “t” is the level of confidence/Alpha level (value for the selected alpha level-indicates the level of risk the re- searcher is willing to take so that true margin of error may exceed the acceptable margin of error); (p) (q)-“p” and “q” are the estimates of the variance of the population; estimate of variance calculated as = 0.25 (maximum possible proportion (0.5) ∗1-maximum possible proportion (0.5) produces maximum possible sample size); and “d” is acceptable margin of error for proportion being estimated = 0.05 (5 %) (Error researcher is willing to take). If this formula is applied to the study and an alpha level of 1.65 (0.10), the estimated variance of 0.5 and an error level of 0.05 were used, the formula would be as follows (Eq. (2)):

(2) $$\begin{array}{ccc} & & N_{0}=\frac{{\left(1.64\right)}^{2*}\left(0.5\right)\left(0.5\right)}{{\left(0.05\right)}^{2}}\\ & & N_{0}=272 \end{array}$$

However, when “N_0_” exceeds 5 % of the population (127) Cochran's correctional formula should be applied:

(3) $$N_{1}=\frac{N_{0}}{1+\left(N_{0}/\text{population}\right)}$$

(4) $$\begin{array}{ccc} N_{1} & = & \frac{272}{1+\left(272/127\right)}\\ & = & 86 \end{array}$$

A sample size of 86 would therefore be sufficient for research purposes. The data collection process, however allowed the inclusion of 93 women and 61 men (partners were also interviewed using the same questionnaire as the one used for the women). Table 3 summarizes the distribution of participants interviewed per district municipality.

**Table 3:** Distribution of participants interviewed per local district municipality.

District	Local district Municipality	Number of female farmers on list	Interviewed Females	Interviewed males	Total interviewed
Frances Baard District of the Northern Cape	Dikgatlong	45	22	17	39
	Magareng	21	18	13	31
	Phokwane	54	37	20	57
	Sol Plaatje	7	16	11	27
	Total	127	93	61	154

## Limitations

The research focussed on the different attributes of the Abbreviated Women's Empowerment in Agriculture Index. However, the ability and capacity to adapt to change are not confined to the domains and variables of this index. Other factors such as individual characteristics, the current unemployment rate in the country, as well as interpersonal relations associated with the social and cultural sphere in the community also influence empowerment [8]. These factors are not accounted for in the quantitative research conducted.

## Ethics Statement/Institutional Review Board Statement

Ethical approval/ ethical clearance certificate was obtained from the University of the Free State Research General/Human Research Ethics Committee (GHREC). The ethics clearance number: UFS-HSD2019/1191. As this study involved humans (female livestock farmers), the major ethical issues that are most relevant and were addressed are: informed consent, confidentiality, fatigue, and honesty in reporting research findings. All these were considered and addressed accordingly. The farmers were all informed of the study objective and participation was strictly voluntary. Respondents had a choice to pull out of the study any time, and breaks were taken whenever respondents felt tired; please see the ethics letter of approval attached.

## Credit Author Statement

Lindie von Maltitz and Yonas T. Bahta significantly contributed to the present manuscript's preparation. Lindie von Maltitz performed the conceptualization, data collection, and data analysis, and contributed to drafting the article. Yonas T. Bahta. was the supervisor, project leader, and administrator, aided with constructive comments towards the study.

## Data Availability

Drought reslience of female smallholder farmers in the Northern Cape data (Original data)
